# Frequency of respiratory virus infections and next-generation analysis of influenza A/H1N1pdm09 dynamics in the lower respiratory tract of patients admitted to the ICU

**DOI:** 10.1371/journal.pone.0178926

**Published:** 2017-06-07

**Authors:** Antonio Piralla, Francesca Rovida, Alessia Girello, Marta Premoli, Francesco Mojoli, Mirko Belliato, Antonio Braschi, Giorgio Iotti, Elena Pariani, Laura Bubba, Alessandro R. Zanetti, Fausto Baldanti

**Affiliations:** 1 Molecular Virology Unit, Microbiology and Virology Department, Fondazione IRCCS Policlinico San Matteo, Pavia, Italy; 2 Section of Surgery and Anesthesiology, Unit of Anesthesia, Intensive Care and Pain Therapy, Department of Clinical, Surgical, Diagnostic and Pediatric Sciences, University of Pavia, Italy; 3 Department of Anesthesia and Intensive Care, Fondazione IRCCS Policlinico San Matteo, Pavia, Italy; 4 Department of Biomedical Sciences for Health, University of Milan, Milan, Italy; 5 European Programme for Public Health Microbiology Training (EUPHEM), European Centre for Disease Prevention and Control (ECDC), Stockholm, Sweden; 6 Department of Clinical, Surgical, Diagnostic and Pediatric Sciences, University of Pavia, Pavia, Italy; Kliniken der Stadt Köln gGmbH, GERMANY

## Abstract

Recent molecular diagnostic methods have significantly improved the diagnosis of viral pneumonia in intensive care units (ICUs). It has been observed that 222G/N changes in the HA gene of H1N1pdm09 are associated with increased lower respiratory tract (LRT) replication and worse clinical outcome. In the present study, the frequency of respiratory viruses was assessed in respiratory samples from 88 patients admitted to 16 ICUs during the 2014–2015 winter-spring season in Lombardy. Sixty-nine out of 88 (78.4%) patients were positive for a respiratory viral infection at admission. Of these, 57/69 (82.6%) were positive for influenza A (41 A/H1N1pdm09 and 15 A/H3N2), 8/69 (11.6%) for HRV, 2/69 (2.9%) for RSV and 2/69 (2.9%) for influenza B. Phylogenetic analysis of influenza A/H1N1pdm09 strains from 28/41 ICU-patients and 21 patients with mild respiratory syndrome not requiring hospitalization, showed the clear predominance of subgroup 6B strains. The median influenza A load in LRT samples of ICU patients was higher than that observed in the upper respiratory tract (URT) (p<0.05). Overall, a greater number of H1N1pdm09 virus variants were observed using next generation sequencing on partial HA sequences (codons 180–286) in clinical samples from the LRT as compared to URT. In addition, 222G/N/A mutations were observed in 30% of LRT samples from ICU patients. Finally, intra-host evolution analysis showed the presence of different dynamics of viral population in LRT of patients hospitalized in ICU with a severe influenza infection.

## Introduction

While, severe bacterial community-acquired pneumonia is a well-known problem, severe viral infections might be underestimated due to low awareness. On the other hand, the introduction of molecular diagnostic methods has significantly improved the epidemiological investigation of viral pneumonia in the ICU in recent years with increased potential for detection of a wide array of viruses. In fact, in recent studies, viruses accounted for 20–50% of total infections in patients admitted to the intensive care unit (ICU) with severe respiratory distress [1–4]. In addition, the emergence of new respiratory viruses such as human coronavirus (hCoV) SARS, hCoV-MERS and influenza A/H1N1pdm09, has further underscored the role of viruses in severe respiratory infections. Still, the role of viral agents other than influenza such as rhinovirus (HRV), parainfluenza viruses 1–4 (hPIV1-4), respiratory syncytial virus (RSV) and hCoV which are frequently detected in respiratory samples of ICU patients, is not fully appreciated [1,2].

During the 2014–2015 influenza season, an increased number of laboratory-confirmed influenza admissions to ICUs (and deaths), as compared to previous seasons was observed in the Northern Hemisphere [5]. In general, the emergence of inherently more virulent viruses during the course of a given season should not be overlooked [6]; while, different host factors responsible for the severe outcome of influenza infection have been suggested (i.e. pregnancy and obesity) [7]. More specifically as observed during the 2009 influenza virus (A/H1N1pdm09) pandemic, only a few genetic changes may increase transmissibility, replicative efficiency and tissue tropism range [8–12]. For instance, single amino acid changes (222G/N) in the HA gene are associated with increased virus replication in the lower respiratory tract (LRT) and worse clinical outcome [8–12]. However, it is still unclear how these mutations emerge, evolve, consolidate and/or disappear in the context of dynamic viral population changes during the infection.

The aims of this study were: i) to describe the frequency of respiratory viruses in patients admitted to the ICU during a winter-spring season in a multicenter prospective observational study; ii) to monitor the presence of polymorphisms at position 222 in the HA gene of influenza A/H1N1pdm09 strains and iii) to investigate the dynamics of an influenza virus population in the LRT of ICU patients using both Sanger and next generation sequencing (NGS).

## Materials and methods

### Study design

From December 1 2014 to April 30 2015, nasal swabs (NS), nasopharyngeal aspirates (NPA), bronchoalveolar lavage (BAL) and broncho aspirates (Brasp), collected from patients with acute respiratory failure requiring ICU admission in Lombardy (10 million inhabitants), were prospectively analyzed as part of a Regional Influenza Surveillance Plan. Analyses were centralized at the Molecular Virology Unit, Fondazione IRCCS Policlinico San Matteo, Pavia or at the Department of Biomedical Sciences for Health, University of Milan. Severe respiratory syndrome was defined as acute onset (≤1 week) respiratory failure, with hypoxemia (pO_2_/FiO_2_ ratio <300 mmHg while on positive end-expiratory pressure (PEEP) or noninvasive CPAP ≥5 cmH_2_O) and bilateral opacities at chest imaging [13].

All samples positive for influenza A/H1N1pdm09 virus were analyzed for the presence of polymorphisms at position 222 of the HA gene. In addition, a group of A/H1N1pdm09-positive patients with mild respiratory syndrome not requiring hospitalization were included in the study as controls.

### Ethics statement

This retrospective study was performed according to the the Regional Surveillance and Preparedness Plan (DGR IX/1046, 22 Dec. 2010 and DGR 5988, 30 Jun 2011) and to the guidelines of the Institutional Review Board on the use of biological specimens for scientific purposes in keeping with Italian law (art.13 D.Lgs 196/2003) and was approved by the Ethics Commitee of Fondazione IRCCS Policlinico San Matteo in Pavia, Italy. Diagnostic and clinical management of patients admitted to hospitals in the Lombardy Region with severe and moderate influenza like illness (ILI) included prospective influenza A detection, subtyping and sequencing. The latter two were centralized at two Regional Reference Laboratories. Mild respiratory infection specimens were collected by sentinel practitioners and anonymously analyzed at the reference laboratory in Milan, according to the National Surveillance Plan (Influnet). Informed consent for Influenza A genotyping was not required since patients with severe and mild ILI were included in the Regional diagnostic and clinical management protocol. Data were anonymously analyzed according to the Regional Surveillance and Preparedness Plan; while, mild ILI samples were collected and anonymously analyzed in the framework of the National Surveillance Plan (Influnet).

### Respiratory virus detection and other microbiological investigations

Nucleic acids from respiratory samples were extracted using the QIAsymphony^®^ instrument with the QIAsymphony^®^ DSP Virus/Pathogen—Midi Kit (Complex 400 protocol) according to the manufacturer’s instructions (QIAGEN, Qiagen, Hilden, Germany). A panel of laboratory-developed real-time RT-PCR or real-time PCR [14,15] were used; these are able to detect and quantify the following viruses: influenza virus A and B (including subtype determination), HRV, hPIV3, RSV types A and B, hCoV-OC43, -229E, -NL63, and -HKU1, and human metapneumovirus (hMPV). Bacterial and fungal cultures were performed, with the use of standard techniques, on quantified BAL and Brasp specimens.

### HA Sanger sequencing and phylogenetic analysis

The complete influenza A/H1N1pdm09 HA gene was amplified directly from clinical specimens using the SuperScriptIII One-Step RT-PCR amplification kit (Invitrogen, Carlsbad, USA) and specific primers (available upon request). Purified PCR products were sequenced using the BigDye Terminator Cycle-Sequencing kit (Applied Biosystems, Foster City, USA) in an ABI Prism 3130xl Genetic Analyzer (Applied Biosystems, Foster City, USA). Sequences were assembled using the Sequencher software, version 4.6 (Gene Codes Corporation, Ann Arbor, USA). Nucleotide alignments were constructed using the ClustalW program embedded in the MEGA version 5 software [16]. Phylogenetic trees were generated by means of the Maximum likelihood method with the Kimura 3-parameter as an evolutionary model using MEGA version 5 software [16]. A bootstrap analysis with 1,000 replicates was performed. Sanger sequences originated in this study have been submitted to GenBank database with accession numbers KY345117-KY345168.

### NGS amplicon

Primers targeting the influenza A HA gene spanning codons 180–286 were used to amplify the region containing codon 222, as previously reported [17]. For synthesis of cDNA, the reaction was carried out using the Transcriptor High Fidelity cDNA synthesis kit (Roche Diangnostics, GmBH) and 5 ul of extracted RNA. Amplifications were performed using the FastStart High-Fidelity PCR System kit (Roche Applied Science) with the following reagent mix: 10 μl of extracted DNA, 5 μl of 10x buffer, 0.25 μl of each primer (10 uM) HA222-for 5’-CACCATCCATCTACTAGTGCTGAC-3’ and HA222-rev 5’-ATAGCACCYTTGGGTGTCTG-3’, 1 μl of PCR grade nucleotide mix (200μM each dNTP), 0.5 μl of FastStart High-Fidelity enzyme blend (5U/μl) and 28.5 μl PCR grade water. The cycling conditions for the amplification were as follows: 95°C for 2 min; 45 cycles of 95°C for 30s, 60°C for 30s, and 72°C for 1 min, with a final extension cycle at 72°C for 5 min.

### Library preparation, emulsion and sequencing

Amplicons were purified with Agencourt AMPure XP magnetic beads (Beckman Coulter, High Wycombe, UK) and quantified with the Quant-iT PicoGreen dsDNA Assay Kit (Invitrogen, Life Technologies, Paisley, UK) with a TBS-380 Mini-Fluorometer (Turner Biosystems, Sunnyvale, CA). A dilution of 1×10^9^ molecules/μl was performed for each amplicon and then pooled in an equimolar concentration. The mixture was further purified with Agencourt AMPure XP magnetic beads (Beckman Coulter, High Wycombe, UK) and diluted to 1×10^6^ molecules/ul. The purified pool was clonally amplified by emulsion PCR (emPCR), following the emPCR Amplification Manual for 454 GS Junior Titanium. A total of 500,000 enriched beads were deposited into a PicoTitrePlate (PTP) device and sequenced using the GS Junior 454 system (Roche Diagnostics, West Sussex, UK).

### Analysis of NGS data

Sequencing data were obtained as standard flowgram format (SFF) files from the GS run processor after quality-control filtering and read trimming using the manufacturer's default parameters (see 454 Sequencing System software manual version 2.5). The SFF data files were further analyzed using the GS Amplicon Variant Analyzer (AVA) version 2.7 (Roche 454 Life Sciences, Branford, CT). The reads were trimmed using the multiplex identifiers (MIDs) for each sample, and mapped using the reference FASTA HA sequences of the California/07/2009 strain (AY6894). The identification of variants was made using the default AVA settings. We considered all changes whose frequency was at least 0.50% (i.e. three times the insertion error rate) as true variability; this was the highest frequency for procedural/experimental errors. A variant was defined as a virus that had at least one mutated nucleotide in given sites of the nucleotide sequence analyzed.

### Statistical analyses

Continuous variables (i.e. viral load, variant numbers) were compared using the Mann-Whitney U test for independent non-parametric data. The Spearman rank correlation coefficient was used for the correlation analysis of non-parametric data. All of the analyses were two tailed, and performed using *GraphPad Prism* version 5 (GraphPad Software Inc., CA, USA); *p*-values of ≤0.05 were considered statistically significant.

## Results

### Patients and samples

**Fig 1:**
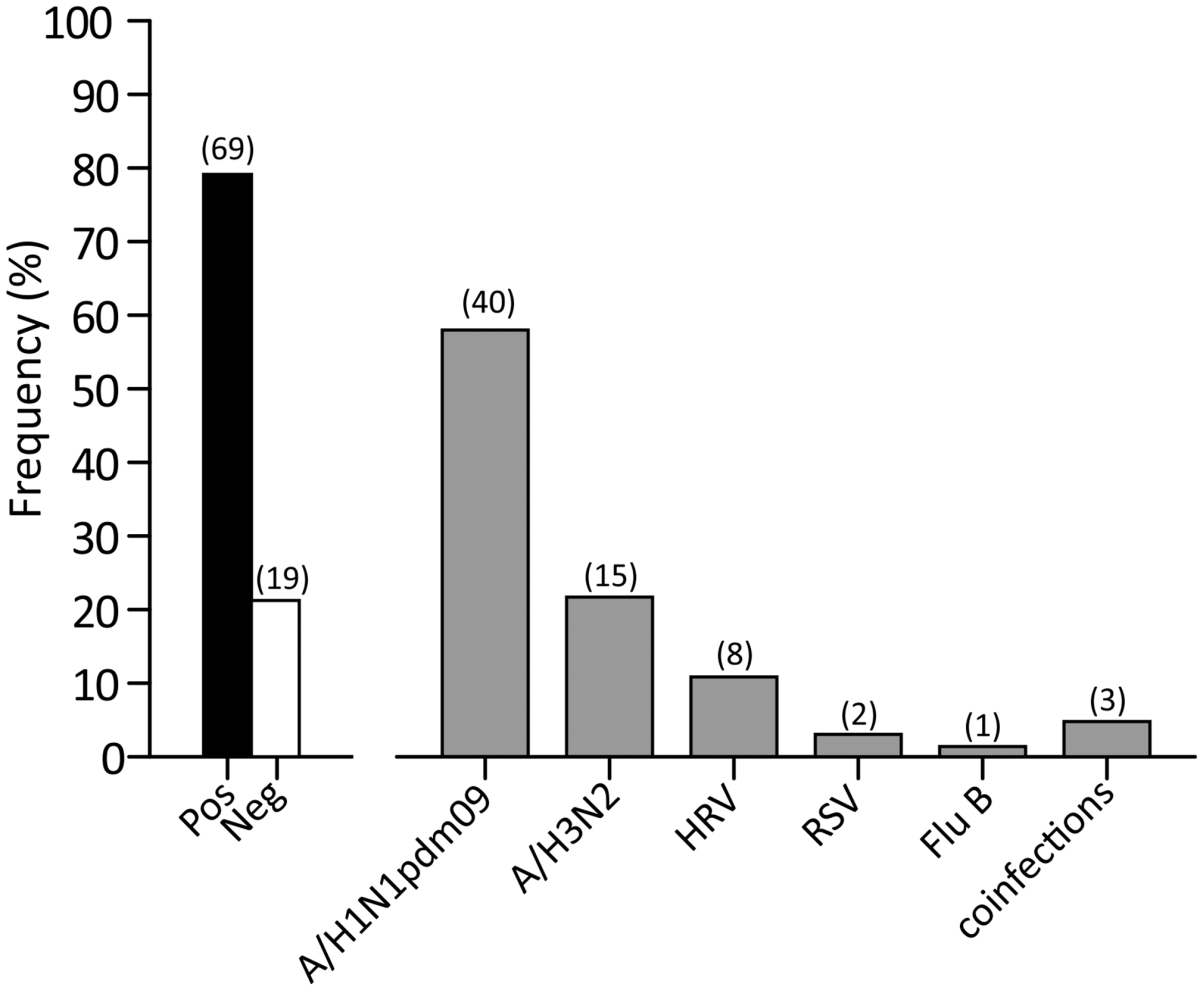
Frequency distribution of respiratory viruses. The absolute number of positive patients is reported in brackets.

A total of 150 respiratory samples from 88 patients admitted to ICUs with severe respiratory syndrome were analyzed. Of these, 90/150 (60.0%) were URT specimens (NS or NPA), while 60/150 (40.0%) were LRT specimens (BAL or Brasp). Sixty-nine out of 88 (78.4%) patients were positive for a respiratory viral infection, while 19/88 (21.6%) were negative (Fig 1). In 66/69 (95.6%) of the positive episodes, a single virus was observed, while in 3/69 (4.4%) episodes dual viral infections were detected (Fig 1). Influenza A was the most frequent virus observed (57/69, 82.6%). In 41/57 (71.9%) patients, subtype A/H1N1pdm09 (40 single and 1 co-infection) was detected, while in 16/57 (28.1%) patients a subtype A/H3N2 (15 single and 1 co-infection) was detected. HRV was detected in 8/69 (11.6%) patients, RSV in 2/69 (2.9%) and influenza B in 1/69 (1.4%). In three patients, dual viral infections were observed: influenza A/H1N1pdm09 and HRV (1 pt), influenza B and RSV (1 pt) and influenza A/H3N2 and hCoV (1 pt). For a subset of patients (23/88, 26.1%), additional information on bacterial/fungal co-infections were available. Among them, 2/23 (8.7%) had a coinfections with influenza A/H1N1pdm09 and *Candida spp*, 1/23 (4.3%) with A/H1N1pdm09 and *Aspergillus spp*, 1/23 (4.3%) with influenza A and *K*. *pneumoniae*, while 19/23 (82.7%) were negative (data not shown).

### Influenza A/H1N1pdm09 viral load comparison: URT vs LRT

Among the 41 ICU patients, paired samples (URT and LRT) were available for 10 (24.4%), from 28 (68.3%) only URT specimens were available and from 3 (7.3%) only LRT specimens were available. The median viral load in LRT samples (n = 13) was higher than that observed in the URT samples (n = 38) (2.8x10^6^ *vs* 2.5x10^4^ RNA copies/ml; p<0.05). Similarly, the median viral load in URT samples of control patients was slightly higher than that observed in the URT of ICU patients, although this difference did not reach significance (p>0.05). When restricting the analysis to the 10 ICU patients with paired samples, influenza load was significantly higher in LRT secretions (median 9.5x10^6^ RNA copies/ml, range 9x10^4^-3.8x10^8^) compared to URT samples (median 3.9x10^3^ RNA copies/ml, range 0–1.1x10^9^; p<0.05). In addition, the median Log10 difference between LRT and URT samples was +2.3 Log10 copies/ml ranging from -2.7 to +7.5 Log10 RNA copies/ml. Interestingly, in two cases, the URT samples were negative in the presence of high viral load in the LRT.

### Phylogenetic and sequence analyses

**Fig 2:**
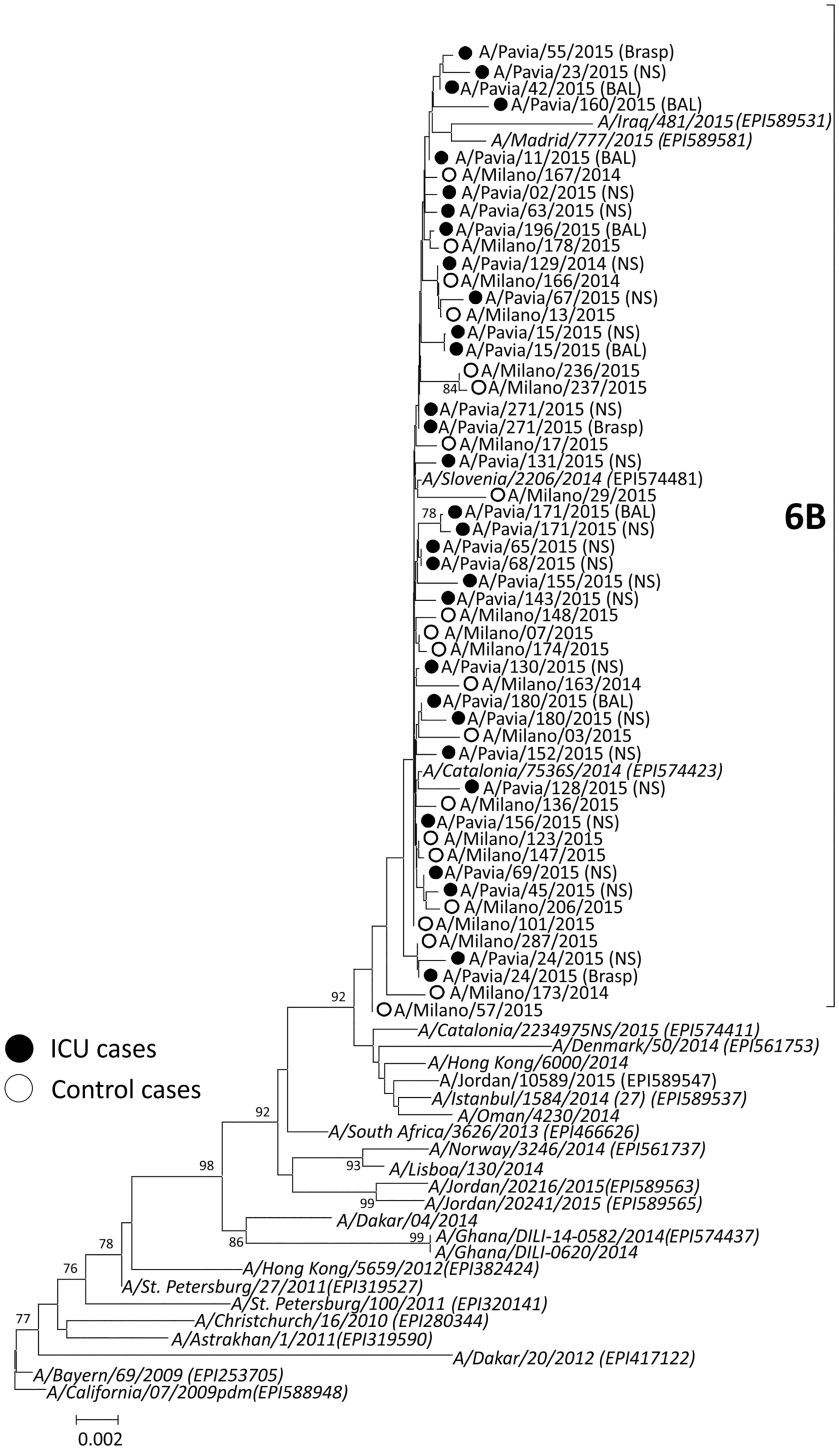
Phylogenetic comparison of influenza A(H1N1)pdm09 HA gene sequences. Reference sequences of strains circulating in 2009–2015 are presented in italics (GISAID numbers are provided). Influenza strains of patients with severe infection are reported with a black circle and strains from patients with a mild infection are reported with a white circle. Bootstrap values are given at nodes, and the scale bar is given in numbers of substitutions per site.

A total of 51 (36 NS and 13 BAL or Brasp) respiratory samples from 41 H1N1pdm09-positive ICU patients as well as 21 NS from patients with mild respiratory syndrome were analyzed by both Sanger and NGS sequencing. Complete HA sequencing was performed on baseline samples from 26/41 (63.4%) ICU and 21/21 (100.0%) control patients. No sequencing data were obtained in respiratory samples of 15/41 (31.7%) ICU patients due to low viral load. Of these, 14/15 (93.3%) sequence-negative samples were URT specimens. The overall nucleotide identity between HA sequences from this study and the reference vaccine strain (A/California/07/2009) ranged from 97.6% to 98.4%. All of the influenza A/H1N1pdm09 strains belonged to subgroup 6B, as observed worldwide (Fig 2). Subgroup 6B was characterized by constitutional K163Q, A256T, K283E and E374K polymorphisms. Additional amino acid polymorphisms were sporadically observed in HA sequences of a few strains. In detail, the A/Pavia/160/2015 strain carried the G39R and S74R changes, A/Pavia/24/2015 strain the S84R change, A/Pavia/45/2015 the T232I change, A/Pavia/23/2015 the P236T change and A/Pavia/180/2015 the E283K change.

Among ICU patients, 22/26 (84.6%) influenza-sequenced strains carried the wild-type 222D codon, while four (15.7%) strains carried mixtures at position 222 (Table 1). In detail, two patients had a mixture of 222D/N/G, one the 222D/N/A mixture and the other 222D/N. All of these mixtures were only identified in LRT samples.

**Table 1:** Distribution of 222 polymorphisms in 10 paired upper and lower respiratory tract samples and 3 lower respiratory samples analyzed with Sanger and next generation sequencing (NGS).

#	Strain	Sample	FluA load (copies/ml)	No. reads	222 polymorphisms		No. variants
					NGS (%)	Sanger	
1	A/Pavia/11/2015	NS	negative	NA	-	-	-
		BAL	3.8x10^7^	7566	D (34.59), N (38.53), G (26.88)	D/N/G	30
2	A/Pavia/15/2015	NS	5.9x10^4^	3264	D (100)	D	7
		BAL	1.9x10^5^	4725	D (100)	D	17
3	A/Pavia/42/2015	NS	1.8x10^2^	ND LVL	-	-	-
		BAL	1.2x10^8^	5593	D (38.98), N (58.1) A (2.91)	D/N	12
4	A/Pavia/171/2015	NS	5.2x10^5^	6493	D (100)	D	8
		BAL	6.4x10^8^	5993	D (100)	D	16
5	A/Pavia/180/2015	NS	1.7x10^8^	5155	D (100)	D	11
		BAL	2.9x10^5^	6463	D (100)	D	11
6	A/Pavia/24/2015	NS	7.1x10^3^	ND, LVL	-	-	-
		Brasp	1.3x10^6^	7969	D (61.06), N (21.23), G (17.71)	D/N/G	21
7	A/Pavia/160/2015	NS	negative	NA	-	D	-
		BAL	5.3x10^5^	7092	D (100)	D	8
8	A/Pavia/271/2015	NS	1.0x10^9^	6818	D (100)	D	4
		Brasp	2.7x10^9^	6847	D (100)	D	16
9	A/Pavia/247/2015	NS	4.5x10^2^	ND, LVL	-	-	-
		Brasp	9.0x10^4^	4798	D (100)	D	3
10	A/Pavia/55/2015	NS	1.1x10^4^	NA	-	-	-
		Brasp	1.7x10^6^	5473	D (34.04), N (65.96)	D/N	18
11	A/Pavia/25/2015	BAL	5.3x10^4^	428	D (100)	D	2
12	A/Pavia/196/2015	BAL	2.8x10^6^	4895	D (100)	D	7
13	A/Pavia/267/2015	BAL	1.8x10^2^	ND, LVL	-	-	-

NS, nasal swab; BAL, bronchoalveolar lavage; Brasp, broncho aspirate; NA, not applicable, ND, not done; LVL, low viral load

### Influenza A/H1N1pdm09 variant analysis

NGS was used to analyze the dynamic of Influenza A/H1N1pdm09 population in partial HA region, spanning from 180 to 286 codons. NGS analysis was successfully performed in 31/41 (75.6%) ICU and 21/21 (100.0%) control patients. In 10/41 (31.7%) ICU patients, no NGS data were obtained for URT samples due to the low viral load. A total of 409072 reads were obtained with the NGS platform, and the average of amplicons length was 341 nt. A median of 5506 (range 1966–13083) sequence reads were obtained per sample, which allowed detection and quantification of minority mutations with a cut-off value of 0.50%.

**Fig 3:**
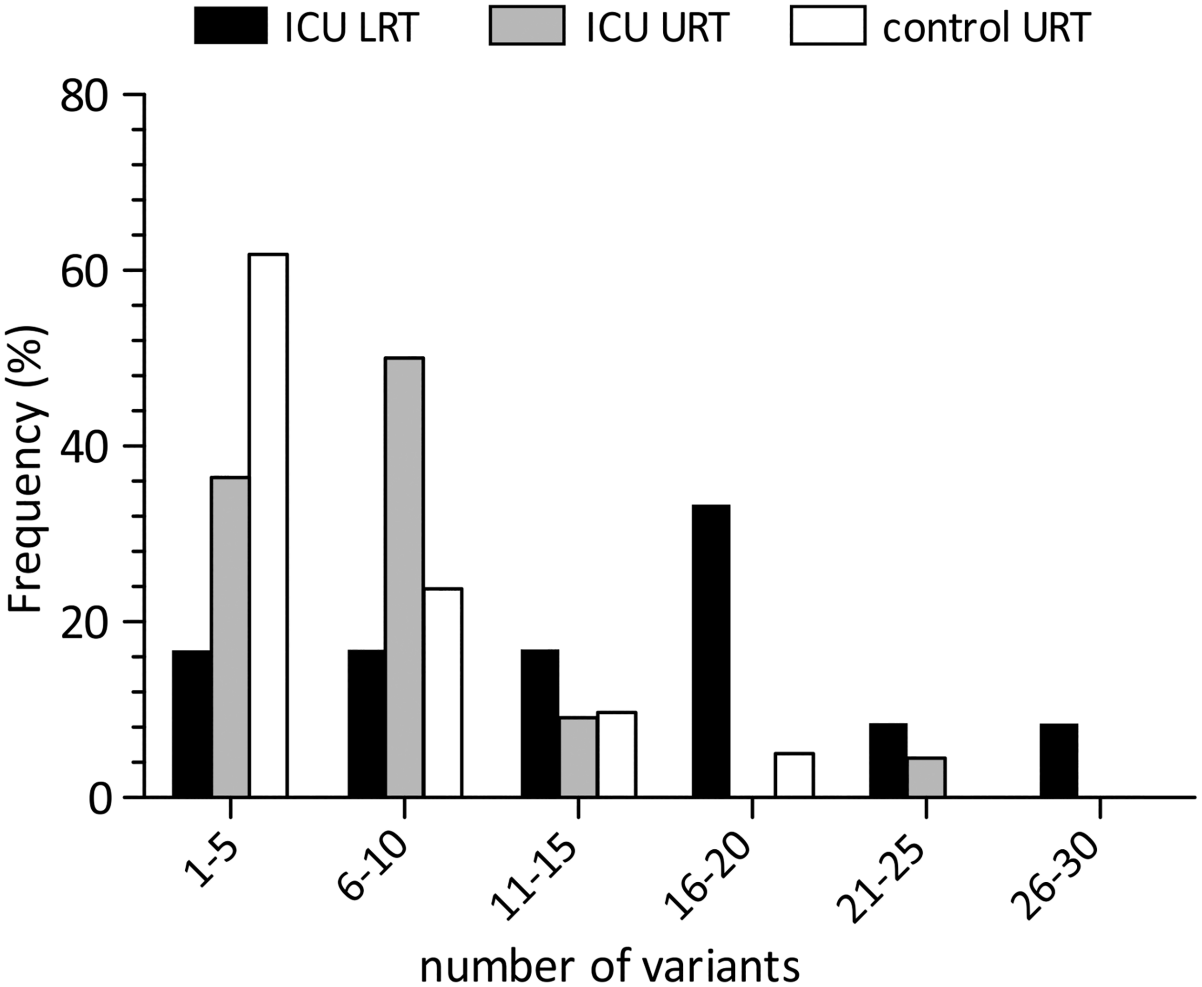
Distribution of the number of variants in both URT and LRT samples of ICU patients and URT samples of patients with mild respiratory syndrome.

Among ICU patients (n = 41), the number of variants in the LRT samples (median 14, range 2–30) was significantly higher than that observed in URT samples (median 7, range 2–21; p<0.05)(Fig 3). Similarly, the number of variants observed in URT samples of ICU patients (median 7, range 2–21) was higher than that observed in URT samples of control patients (median 4, range 2–16; p<0.05). Among ten patients with paired samples (#1–10 in Table 1), the number of variants was performed in paired URT and LRT samples for 4/10 (40.0%) patients, while in 6/10 (60.0%) patients it could only be performed in LRT samples, due to the low viral load in URT samples (Table 1). For three additional patients (#11–13, Table 1), the number of variants was determined only in LRT samples.

Overall, mutations (G/N/A) at codon 222 were observed in 4/13 (30.8%) LRT samples (Table 1). In these patients, a number of mixed variants (as also suggested by Sanger mixed electropherograms) were detected. All H1N1pdm09 strains detected in the URT of ICU (n = 22) and control (n = 21) patients carried a 222D polymorphism at a 100% frequency. Only in one patient, did NGS identify a 222A change with a frequency lower than 5.0% in a LRT sample. Of note, samples in which the NGS analysis identified only a 222D polymorphism showed an overall lower number of variants (median 9.5, range 2–17 variants) as compared to samples with mixed variants at position 222 (median 19.5, range 12–30 variants; p<0.05).

### Longitudinal analysis in LRT infections

**Fig 4:**
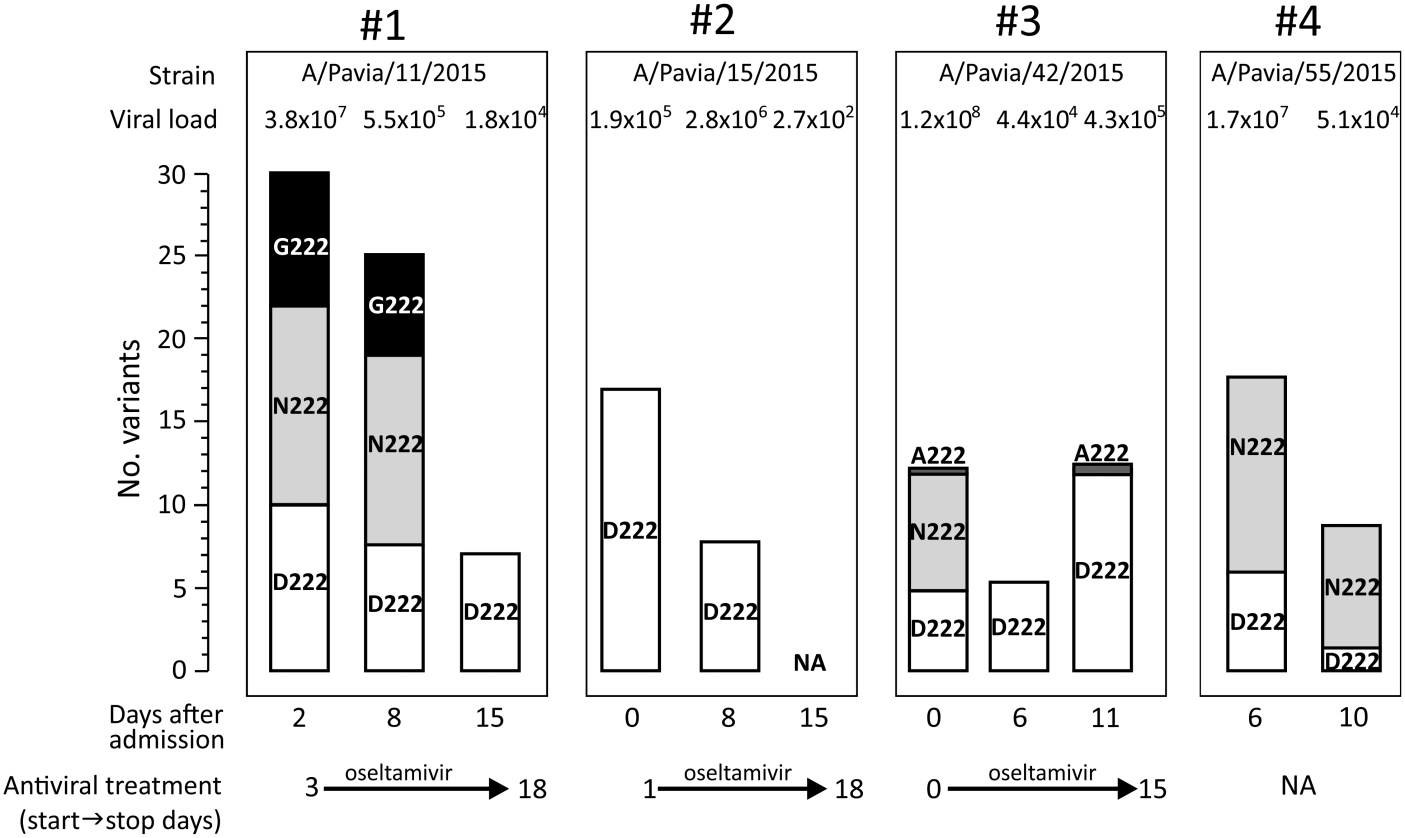
Frequencies of 222 polymorphisms are displayed as a stacked histogram for four patients with sequential lower respiratory tract samples. The number of variants observed and the corresponding viral load are reported above each histogram, while the time after admission and the antiviral treatment period are reported below each histogram. NA, not available.

Follow-up LRT samples were collected in a limited number of patients (n = 4). In these cases, a longitudinal NGS analysis of the viral population was possible. These four patients received anti-influenza treatment during the analyzed period as reported in Fig 4. At baseline, in 3/4 cases (75.0%), a mixture of mutations at codon 222 was observed (Fig 4). In samples from patients #1 and #2, viral load and the number of variants decreased simultaneously and only the wild-type amino acid (222D) was detected 13 and 8 days after admission. In samples from patient #3, an initial decrease in viral load and number of variants was associated with the presence of wild-type amino acid 222D. However, a sudden increase in viral load and number of variants was associated with the re-emergence of 222A at a low frequency. From patient #4, only LRT samples at days 6 and 10 after onset of symptoms were available and the decrease in both viral load and number of variants was not associated with significant changes in the viral population at position 222.

## Discussion

In the present prospective observational study, the incidence of respiratory viruses was investigated in respiratory samples of patients admitted to 16 ICUs during the 2014–2015 influenza season. Influenza viruses were the most commonly detected pathogens. The second most identified virus was HRV with a frequency close to 10%, in keeping with a large prospective study describing the aetiology of community-acquired pneumonia in the US [18]. The clinical relevance of non-influenza viral infections in ICU patients remains controversial [19–21]. However, the impact of non-influenza viruses appears more important than previously thought; thus, these viruses should be included in the diagnostic panel [3,22].

In this study, A/H1N1pdm09 strains outnumbered A/H3N2 at a ratio approaching 3:1 in patients admitted to ICUs in Northern Italy. This finding is in contrast with the epidemiological scenario observed by sentinel sources of the World Health Organization and National Influenza Center. In fact, a predominance of influenza A/H3N2 strains in Europe [5] and Italy (http://www.iss.it/binary/fluv/cont/Rapporto_2014_2015.pdf) was observed during the same period.

Among ICU patients, the median influenza load in LRT samples was significantly higher than that observed in URT samples in the same patient group, in keeping with our previous observations [8,10]. The difference between viral loads was more evident in paired samples where in all cases except one, viral load was higher in the LRT than URT. Interestingly, in almost 20% of these patients the viral load in the URT was undetectable in the presence of high viral load in the LRT. It is reasonable to hypothesize that in severe infections, the initial replication of influenza in the URT is followed by the spread of influenza in the LRT [23]. At ICU admission, usually several days after the onset of clinical symptoms, the clinical samples are collected for the laboratory diagnosis. At this moment, the influenza virus might have been already cleared from the URT by the immune system, while in the LRT the viral shedding persists[23]. Therefore, our findings underline the importance of appropriate sample collection, especially in patients with severe infection [23,24].

Considering the D222G/N mutations in the HA gene of influenza A/H1N1pdm09 strains, which are strictly associated with increased clinical severity [9,25–27], in this study, the presence of polymorphisms at position 222 of A/H1N1pdm09 strains was assessed by both Sanger and NGS sequencing in patients with severe infections and in patients (not hospitalized) with a mild respiratory syndrome. All of the A/H1N1pdm09 strains belonged to the influenza clade circulating in Europe during the period of sample collection [28].

Intra-host viral diversity was assessed using NGS in respiratory samples by determining the number of H1N1pdm09 variants. Overall, a greater number of H1N1pdm09 virus variants were observed in the LRT, suggesting different viral kinetic patterns in the URT, as compared to LRT of patients with severe influenza infections. The 222G/N mutations known to be associated with increased clinical severity were observed in 30% of LTR samples of ICU patients. In URT samples (ICU and control patients) only the wild type 222D polymorphism was observed. The frequency of 222G/N changes was in keeping with our previous observations and other reports [8,10,17,29,30]. Although our data confirm the association between 222G/N mutations and disease severity, over 70% of ICU patients showed no specific HA mutations among viral populations in the lung. Therefore, it is reasonable to hypothesize that more than one virulence determinant is involved in severe influenza infections. Nevertheless, in these patients, comorbidities or per-existing host genetic factors may have contributed to disease severity [31].

It has been demonstrated both *in vitro* and *in vivo* that strains carrying 222D bind α2–6 sialic acid more efficiently, whereas 222G/N strains have broader tropisms for both α2–6 and α2–3 sialic acids and therefore replicate more efficiently in the LRT [11,12,30]. In keeping with this finding, in our series, 222G/N populations were observed only in LRT samples. Unfortunately, no information regarding the quasispecies population of the URT could be obtained due to low virus load. Viral diversity, evaluated in sequential LRT samples, showed several patterns of population evolution. On the basis of these findings and, due to the limited number of cases, no major conclusions can be drawn on viral population dynamic in the LRT.

This study has some limitations: i) the frequency of different respiratory viruses could be biased by the role of our center as a reference laboratory for the diagnosis and confirmation of severe influenza-like illness; ii) the quasispecies analysis focused only on codon 222, while complete genome sequencing would have provided more exhaustive information and iii) in a portion of the ICU patients, only upper respiratory samples were available and therefore the diagnosis was performed on a partially biased group of samples.

In conclusion, this study showed the presence of mutations associated with increased clinical severity (222G/N) in at least 30% of ICU patients with LRT infections. A great number of variants and high viral load were observed in the LRT as compared to URT samples. Finally, intra-host evolution analysis showed the presence of different dynamics of viral population in the LRT of patients hospitalized in ICU with a severe influenza infection.

## References

[1] Choi SH, Hong SB, Ko GB, Lee Y, Park HJ, Park SY, et al Viral infection in patients with severe pneumonia requiring intensive care unit admission. Am J Respir Crit Care Med. 2012; 186(4):325–32. 10.1164/rccm.201112-2240OC 22700859

[2] Wiemken T, Peyrani P, Bryant K, Kelley RR, Summersgill J, Arnold F, et al Incidence of respiratory viruses in patients with community-acquired pneumonia admitted to the intensive care unit: results from the Severe Influenza Pneumonia Surveillance (SIPS) project. Eur J Clin Microbiol Infect Dis. 2013; 32(5):705–10. 10.1007/s10096-012-1802-8 23274861

[3] Hong HL, Hong SB, Ko GB, Huh JW, Sung H, Do KH, et al Viral infection is not uncommon in adult patients with severe hospital-acquired pneumonia. PLoS One. 2014; 9(4):e95865 10.1371/journal.pone.0095865 24752070 PMC3994115

[4] Wansaula Z, Olsen SJ, Casal MG, Golenko C, Erhart LM, Kammerer P, et al Surveillance for severe acute respiratory infections in Southern Arizona, 2010–2014. Influenza Other Respir Viruses. 2016; 10(3):161–9. 10.1111/irv.12360 26590069 PMC4814863

[5] Hammond A, Gusbi N, Sosa P, Fitzner J, Besselaar T, Vandemaelea K, et al Review of the 2014–2015 influenza season in the northern hemisphere. Wkly Epidemiol Rec. 2015; 90(23):281–96. 26050269

[6] Taubenberger JK. The origin and virulence of the 1918 "Spanish" influenza virus. Proc Am Philos Soc. 2006; 150(1):86–112. 17526158 PMC2720273

[7] Van Kerkhove MD, Vandemaele KA, Shinde V, Jaramillo-Gutierrez G, Koukounari A, Donnelly CA, et al Risk factors for severe outcomes following 2009 influenza A (H1N1) infection: a global pooled analysis. PLoS Med. 2011 7;8(7):e1001053 10.1371/journal.pmed.1001053 21750667 PMC3130021

[8] Baldanti F, Campanini G, Piralla A, Rovida F, Braschi A, Mojoli F, et al Severe outcome of influenza A/H1N1/09v infection associated with 222G/N polymorphisms in the haemagglutinin: a multicentre study. Clin Microbiol Infect. 2011; 17(8):1166–9. 10.1111/j.1469-0691.2010.03403.x 20946414

[9] Kilander A, Rykkvin R, Dudman SG, Hungnes O. Observed association between the HA1 mutation D222G in the 2009 pandemic influenza A(H1N1) virus and severe clinical outcome, Norway 2009–2010. Euro Surveill. 2010;15(9). pii: 19498. 20214869 10.2807/ese.15.09.19498-en

[10] Piralla A, Pariani E, Rovida F, Campanini G, Muzzi A, Emmi V, et al Segregation of virulent influenza A(H1N1) variants in the lower respiratory tract of critically ill patients during the 2010–2011 seasonal epidemic. PLoS One. 2011;6(12):e28332 10.1371/journal.pone.0028332 22194826 PMC3237448

[11] Chutinimitkul S, Herfst S, Steel J, Lowen AC, Ye J, van Riel D, et al Virulence-associated substitution D222G in the hemagglutinin of 2009 pandemic influenza A(H1N1) virus affects receptor binding. J Virol. 2010;84(22):11802–13. 10.1128/JVI.01136-10 20844044 PMC2977876

[12] Liu Y, Childs RA, Matrosovich T, Wharton S, Palma AS, Chai W, et al Altered receptor specificity and cell tropism of D222G hemagglutinin mutants isolated from fatal cases of pandemic A(H1N1) 2009 influenza virus. J Virol. 2010; 84(22):12069–74. 10.1128/JVI.01639-10 20826688 PMC2977873

[13] ARDS Definition Task Force., Ranieri VM, Rubenfeld GD, Thompson BT, Ferguson ND, Caldwell E, Fan E, et al Acute respiratory distress syndrome: the Berlin Definition. JAMA. 2012; 307(23):2526–33. 10.1001/jama.2012.5669 22797452

[14] Piralla A, Baldanti F, Gerna G. Phylogenetic patterns of human respiratory picornavirus species, including the newly identified group C rhinoviruses, during a 1-year surveillance of a hospitalized patient population in Italy. J Clin Microbiol. 2011; 49(1):373–376. 10.1128/JCM.01814-10 21068279 PMC3020431

[15] Piralla A, Lunghi G, Percivalle E, Viganò C, Nasta T, Pugni L, et al FilmArray^®^ respiratory panel performance in respiratory samples from neonatal care units. Diagn Microbiol Infect Dis. 2014;79(2):183–6. 10.1016/j.diagmicrobio.2014.02.010 24666702 PMC7132758

[16] Tamura K, Peterson D, Peterson N, Stecher G, Nei M, Kumar S. MEGA5: molecular evolutionary genetics analysis using maximum likelihood, evolutionary distance, and maximum parsimony methods Mol Biol Evol. 2011; 28: 2731–2739. 10.1093/molbev/msr121 21546353 PMC3203626

[17] Selleri M, Piralla A, Rozera G, Giombini E, Bartolini B, Abbate I, et al Detection of haemagglutinin D222 polymorphisms in influenza A(H1N1)pdm09-infected patients by ultra-deep pyrosequencing. Clin Microbiol Infect. 2013; 19(7):668–73. 10.1111/j.1469-0691.2012.03984.x 22862843

[18] Jain S, Self WH, Wunderink RG, Fakhran S, Balk R, Bramley AM, et al Community-Acquired Pneumonia Requiring Hospitalization among U.S. Adults. N Engl J Med. 2015; 373(5):415–27. 10.1056/NEJMoa1500245 26172429 PMC4728150

[19] Luchsinger V, Ruiz M, Zunino E, Martínez MA, Machado C, Piedra PA, et al Community-acquired pneumonia in Chile: the clinical relevance in the detection of viruses and atypical bacteria. Thorax. 2013; 68(11):1000–6. 10.1136/thoraxjnl-2013-203551 23783373

[20] Schnell D, Gits-Muselli M, Canet E, Lemiale V, Schlemmer B, Simon F, et al Burden of respiratory viruses in patients with acute respiratory failure. J Med Virol. 2014;86(7):1198–202. 10.1002/jmv.23760 24108695 PMC7167001

[21] van Someren Gréve F, Ong DS, Cremer OL, Bonten MJ, Bos LD, de Jong MD, et al Clinical practice of respiratory virus diagnostics in critically ill patients with a suspected pneumonia: A prospective observational study. J Clin Virol. 2016; 83:37–42. 10.1016/j.jcv.2016.08.295 27567093 PMC7106504

[22] Luyt CE. Virus diseases in ICU patients: a long time underestimated; but be aware of overestimation. Intensive Care Med. 2006; 32(7):968–70. 10.1007/s00134-006-0203-9 16791659 PMC7079854

[23] Lee N, Chan PK, Wong CK, Wong KT, Choi KW, Joynt GM, et al Viral clearance and inflammatory response patterns in adults hospitalized for pandemic 2009 influenza A(H1N1) virus pneumonia. Antivir Ther. 2011; 16(2):237–47. 10.3851/IMP1722 21447873

[24] Ginocchio CC and McAdam AJ. Current best practices for respiratory virus testing. J Clin Microbiol. 2011; 49(9 Suppl): S44–S48. 10.1128/JCM.00698-11 PMCID: PMC3185851

[25] Wedde M, Wahlisch S, Wolff T, Schweiger B. Predominance of HA-222D/G Polymorphism in influenza A(H1N1)pdm09 viruses associated with fatal and severe outcomes recently circulating in Germany. PLoS One. 2013; 8(2):e57059 10.1371/journal.pone.0057059 23451145 PMC3581548

[26] Kuroda M, Katano H, Nakajima N, Tobiume M, Ainai A, Sekizuka T, et al Characterization of quasispecies of pandemic 2009 influenza A virus (A/H1N1/2009) by de novo sequencing using a next-generation DNA sequencer. PLoS One. 2010; 5(4):e10256 10.1371/journal.pone.0010256 20428231 PMC2859049

[27] Resende PC, Motta FC, Oliveira Mde L, Gregianini TS, Fernandes SB, Cury AL, et al Polymorphisms at residue 222 of the hemagglutinin of pandemic influenza A(H1N1)pdm09: association of quasi-species to morbidity and mortality in different risk categories. PLoS One. 2014; 9(3):e92789 10.1371/journal.pone.0092789 24667815 PMC3965456

[28] European Centre for Disease Prevention and Control. Influenza virus characterisation, summary Europe, May 2015. Stockholm: ECDC; 2015 http://ecdc.europa.eu/en/publications/Publications/influenza-virus-characterisation-May-2015.pdf (accessed 23/02/2016).

[29] Wang B, Dwyer DE, Soedjono M, Shi H, Matlho K, Ratnamohan M, et al Evidence of the circulation of pandemic influenza (H1N1) 2009 with D222D/G/N/S hemagglutinin polymorphisms during the first wave of the 2009 influenza pandemic. J Clin Virol. 2011; 52(4):304–6. 10.1016/j.jcv.2011.08.023 21925936

[30] Seidel N, Sauerbrei A, Wutzler P, Schmidtke M. Hemagglutinin 222D/G polymorphism facilitates fast intra-host evolution of pandemic (H1N1) 2009 influenza A viruses. PLoS One. 2014; 9(8):e104233 10.1371/journal.pone.0104233 25162520 PMC4146462

[31] Lee N, Chan PK, Hui DS, Rainer TH, Wong E, Choi KW, et al Viral loads and duration of viral shedding in adult patients hospitalized with influenza. J Infect Dis. 2009; 200(4):492–500. 10.1086/600383 19591575 PMC7110250

